# Thermodynamic Considerations on the Biophysical Interaction between Low-Energy Electromagnetic Fields and Biosystems

**DOI:** 10.3390/membranes14080179

**Published:** 2024-08-22

**Authors:** Umberto Lucia, Giulia Grisolia

**Affiliations:** 1 Dipartimento Energia “Galileo Ferraris”, Politecnico di Torino, Corso Duca degli Abruzzi 24, 10129 Torino, Italy; 2 Dipartimento di Ingegneria dell’Ambiente, del Territorio e delle Infrastrutture, Politecnico di Torino, Corso Duca degli Abruzzi 24, 10129 Torino, Italy

**Keywords:** extremely low-frequency electromagnetic fields (ELF-EMFs), living systems, membrane ions fluxes, bioengineering thermodynamics, biochemical thermodynamics

## Abstract

A general theory explaining how electromagnetic waves affect cells and biological systems has not been completely accepted yet; nevertheless, extremely low-frequency electromagnetic fields (ELF-EMFs) can interfere with and modify several molecular cellular processes. The therapeutic effect of EMFs has been investigated in several clinical conditions with promising results: in this context a better understanding of mechanisms by which ELF-EMF influences cellular events is necessary and it could lead to more extended and specific clinical applications in different pathological conditions. This paper develops a thermodynamic model to explain how ELF-EMF directly interferes with the cellular membrane, inducing a biological response related to a cellular energy conversion and modification of flows across cell membranes. Indeed, energy, irreversibly consumed by cellular metabolism, is converted into entropy variation. The proposed thermodynamic model views living systems as adaptative open systems, analysing the changes in energy and matter moving in and out of the cell.

## 1. Introduction

Extremely low-frequency electromagnetic fields (ELF-EMFs) are defined by the International Telecommunication Union as electromagnetic radiation (radio waves) with frequencies from 3 to 30 Hz. In atmospheric sciences, an alternative definition is usually given, from 3 Hz to 3 kHz. Moreover, United States Government agencies, such as NASA, describe ELF as non-ionising radiation with frequencies between 0 and 3 kHz [1]. The World Health Organization (WHO) have used ELF-EMF to refer to the concept of “extremely low frequency (ELF) electric and magnetic fields (EMF)” [2]. The WHO also stated that at frequencies between 0 and 3 kHz, “the wavelengths in air are very long (6000 km at 50 Hz and 5000 km at 60 Hz), and, in practical situations, the electric and magnetic fields act independently of one another and are measured separately” [2]. In the ELF range (<3 kHz), the electric and magnetic fields can be considered de-coupled: the electric component may barely diffuse in the human body, while the magnetic component may well penetrate the body nearly un-attenuated [3].

ELF-EMFs have been crucial for the development of life on the Earth, contributing to cell division, governing many other bio-processes [4,5,6], e.g., gene expression, protein transcription, and phosphorylation, and influencing cell functions such as proliferation, differentiation, and apoptosis. Many bio-molecular mechanisms were suggested to explain how ELF-EMFs may affect cells and biological systems [4]; nevertheless, numerous biochemical effects were described related to the biological model considered, intrinsic susceptibility/responsiveness of different cell types, duration of the exposure and the selected electromagnetic frequency [7]. Among the described effects, ELF-EMFs have already been well established to affect ion fluxes across the plasma membrane, regulating a lot of membrane-mediated signal transduction processes, especially concerning ${\mathrm{Na}}^{+}$, $K^{+}$, ${\mathrm{Cl}}^{-}$, ${\mathrm{Ca}}^{2+}$, ${\mathrm{Fe}}^{2+}$, ${\mathrm{Mn}}^{2+}$, and ${\mathrm{Mg}}^{2+}$ fluxes [8].

This evidence is of particular interest concerning biochemical and biophysical processes because ${\mathrm{Na}}^{+}$, $K^{+}$, and ${\mathrm{Cl}}^{-}$ ion fluxes regulate membrane electric potential, while ${\mathrm{Ca}}^{2+}$ and ${\mathrm{Mg}}^{2+}$ ion fluxes control protein folding, ${\mathrm{Zn}}^{2+}$ controls ${\mathrm{HCO}}^{3-}$ formation, and ${\mathrm{Fe}}^{2+}$ activates molecular oxygen and works as a catalyst for the generation of reactive oxygen species (ROS) in pathological conditions such as carcinogenesis, inflammation, and perfusion injury. Consequently, the results of ELF use for ion flux control are interesting for possible future therapies in different diseases. Indeed, in human cells and tissues, ELF-EMF effects were tested by using some frequency ranges to evaluate potential clinical applications [9] aiming to ameliorate different pathological conditions.

Moreover, the electrochemical communication between cells is recognised as an essential requirement, and the ELF-EMF can interfere with this communication by interacting with the cell membrane and affecting the mobility of ions. These ions include some metal cations such as ${\mathrm{Zn}}^{2+}$, ${\mathrm{Fe}}^{2+}$, ${\mathrm{Cu}}^{+}$, ${\mathrm{Mg}}^{2+}$, and ${\mathrm{Ca}}^{2+}$, which play a crucial role in cell signalling. Specifically, calcium influx through cell membranes has received attention concerning cell communication and EMF interaction, due to the huge variety of intracellular mechanisms that depend on it. The applied EMF, especially one with low energy, can modify existing signal transduction processes in cell membranes because EMF frequencies in the body are normally extremely low and include, for example, the action potentials of nerves, myocardiocytes, and skeletal muscle tissues [10,11]. For example, a resonant frequency [12,13,14] of calcium-gated channels was applied to cells, causing physiological depolarisation and natural activation of the cells [15,16,17].

Several studies investigated the increase in intracellular ${\mathrm{Ca}}^{2+}$ and the higher activity of voltage-gated calcium channels (VGCCs) after exposure to EMF. These changes were investigated and demonstrated in many cell types by using calcium channel blockers to lower or block the cell signalling changes induced by EMF [18,19,20]. Changes in calcium signalling in response to ELF-EMF exposure were highlighted in mitogen-activated rat thymocytes and the activity of immune cells [21,22]; on rat pituitary cells, a 50 Hz magnetic field was shown to increase intracellular ${\mathrm{Ca}}^{2+}$ concentration [23]. A calcium-dependent mechanism is proposed to explain the observed changes in cell shape, preferential orientation, and migration, after field exposure of mouse embryo fibroblasts [24], and the calcium increase was inhibited by ${\mathrm{Ca}}^{2+}$ channel blocker D-600. Calcium concentration can also be altered in histamine-activated HL-60 human leukaemia cells, after 60 Hz EMF exposure, resulting in a 20–40% increase in intracellular ${\mathrm{Ca}}^{2+}$. The observed effects were reversible, which could mean the absence of permanent structural damages induced by acute 60 min exposure to this EMF on ion channels [25].

A possible connection between ELF-EMF and voltage-gated calcium channels (VGCCs) in cell proliferation and apoptosis has been more recently studied [15] in human neural and rat neuroendocrine cells, showing significantly enhanced proliferation, around 40%, and a related limitation of apoptosis after $H_{2}O_{2}$ treatment in both cell types after exposure. In this paper, they provided direct evidence of EMF enhancing the expression of VGCCs on the plasma membrane of the exposed cells increasing the number of VGCCs, not only the calcium current density.

A decrease in ${\mathrm{Cl}}^{-}$ intracellular concentration is directly linked to the malignant proliferation of cancer [26], while an increase in ${\mathrm{Na}}^{+}$ intracellular concentration generates depolarisation of the cell membrane, with a related consequence on the mitotic activity [26,27,28,29,30,31,32,33,34,35,36,37,38,39,40,41,42,43,44]. On the contrary, hyper-polarisation causes an activation of the ${\mathrm{Ca}}^{2+}$-$K^{+}$ channel, which increases the intracellular ${\mathrm{Ca}}^{2+}$ concentration [26,45,46], pointing out the ${\mathrm{Ca}}^{2+}$-$K^{+}$ channel’s role in the control of the membrane electric potential [26,47]. Last, an increase in ${\mathrm{Na}}^{+}$ concentration inside the cell causes inflammation [48].

The effects of extremely low-frequency electromagnetic fields on biological systems have been the subject of debate for over fifty years [49]. Experimental studies have often obtained contradictory results, making it challenging to draw clear conclusions. As a result, our understanding of the interaction between ELF-EMF and biological systems is still not fully developed, despite its fundamental importance for future applications [3,49].

This paper proposes a thermodynamic approach to ion fluxes to model the effect of ELF-EMFs on cell behaviour, with a particular focus on controlling inflammation related to ion fluxes. The Section 2 introduces the thermodynamic model, developing the biophysical basis of interaction between ELF-EMFs and cells, and highlighting the thermodynamic approach to the mechano-biological behaviour of cells. In the Section 3, we delve into the concept of thermal resonance, exploring the heat exchange between the cell and its environment. Through this biophysical approach, we present a summary of experimental results that serve to validate the introduced thermodynamic model. In the Section 4, we will delve into the evolution of knowledge and the approaches introduced in the last century, emphasising how our approach effectively addresses the demands for experimental repeatability, as well as thermodynamic and biophysical considerations. In the Section 5, we provide some insights into the potential use of ELF-EMFs as a therapeutic approach to complement existing therapies.

## 2. Materials and Methods

To explain the existing confined interactions between EMFs and cell systems, we considered the effects of ELF-EMFs from a thermodynamic perspective; indeed, cells obey thermodynamic laws by transforming and dissipating energy [50] into work and disorder, respectively.

Here, we highlighted cells that respond to ELF interaction by shifting their energy conversion, and their metabolic change increases the entropy generation by changing the cell’s specific functions. Consequently, the partial dissipation of the assimilated energy from metabolites is converted into entropy variation, allowing us to study cells as adaptive thermodynamic engines that convert energy by coupling metabolic and biochemical reactions with transport processes [51], sustained by the mass, energy, and ion fluxes through the cell membrane.

From a thermodynamic viewpoint, a cell can be considered a macroscopic system because of the large number of molecules it contains, as well as the energy concentration and cell temperature. Ion concentration follows the relation [52,53]

(1) $$c_{in}=c_{out}\;\exp\left(-\frac{ZF\;\Delta \phi }{R\;T}\right)$$

where *c* is the molecule number concentration, in and out subscripts mean inside and outside of the membrane, respectively, *T* represents the temperature, R=8.314 J $K^{-1}$ ${\mathrm{mol}}^{-1}$ is the ideal gas constant, and Δϕ represents the membrane electric potential expressed by the Goldman–Hodgkin–Katz equation [52,53]:

(2) $$\Delta \phi =\frac{R\;T}{ZF}\;\log_{10}\left(\frac{P_{{\mathrm{Na}}^{+}}\;{\left[{\mathrm{Na}}^{+}\right]}_{\mathrm{out}}+P_{K^{+}}\;{\left[K^{+}\right]}_{\mathrm{out}}+P_{{\mathrm{Cl}}^{-}}\;{\left[{\mathrm{Cl}}^{-}\right]}_{\mathrm{out}}}{P_{{\mathrm{Na}}^{+}}\;{\left[{\mathrm{Na}}^{+}\right]}_{\mathrm{in}}+P_{K^{+}}\;{\left[K^{+}\right]}_{\mathrm{in}}+P_{{\mathrm{Cl}}^{-}}\;{\left[{\mathrm{Cl}}^{-}\right]}_{\mathrm{in}}}\right)$$

where *Z* is the ion’s valence, *P* is its permeability, [A] means A-ion concentration, and *F* is the Faraday constant. This last relation highlights that the cell membrane potential can be modified by alterations in the one or more ion fluxes. The ion channels and transporters provide different permeability to distinct ions, such as ${\mathrm{Na}}^{+}$, $K^{+}$, ${\mathrm{Ca}}^{2+}$ and ${\mathrm{Cl}}^{-}$. Typical concentrations of the principal ions are [52]

- ${\mathrm{Na}}^{+}$: 150.0 mM extracellular, 15.0 mM intracellular;
- $K^{+}$: 4.5 mM extracellular, 120.0 mM intracellular;
- ${\mathrm{Cl}}^{-}$: 116.0 mM extracellular, 20.0 mM intracellular;
- ${\mathrm{Ca}}^{2+}$: 1.2 mM extracellular, $10^{-4}$ mM intracellular.

Now, considering Equation (1), we can obtain the value of the cell membrane electric potential for the same ions:

- ${\mathrm{Na}}^{+}$: Δϕ=59 mV;
- $K^{+}$: Δϕ=−85 mV;
- ${\mathrm{Cl}}^{-}$: Δϕ=−45 mV;
- ${\mathrm{Ca}}^{2+}$: Δϕ=121 mV.

However, the membrane electric potential can also be evaluated by considering the Nernst equation [52]:

(3) ZFΔϕ=ΔG+2.3RTΔpH⇒ZFdϕ=dG+2.3RTdpH

where *G* is the Gibbs potential, F=96,485.34 A $s^{-1}$ ${\mathrm{mol}}^{-1}$ is the Faraday constant, 2.3 ΔpH is the physiological concentration gradient, and $H^{+}$ is the hydrogen ion which is used by the cells in order to modulate the membrane electric potential by changing the $H^{+}$ concentration. At human standard temperature (37 °C), $k_{B}T/e=RT/F\approx 26.7$ mV.

Concerning the electromagnetic waves, we consider that they generate a radiation pressure [54]:

(4) $$p=\frac{\varepsilon \;E^{2}}{2}=\frac{B^{2}}{2\;\mu }\Rightarrow dp=\varepsilon \;E\;dE=\frac{B}{\mu }\;dB$$

where *E* and *B* are the amplitudes of the electric field and magnetic field components, respectively, μ is the magnetic permeability, and $c=1/\sqrt{\mu_{0}\varepsilon_{0}}\approx 3\times 10^{8}$ m $s^{-1}$ is the velocity of light, with $\varepsilon_{0}=8.854\times 10^{-12}$ A s $N^{-1}m^{-1}$ being the electric permittivity and $\mu_{0}=4\pi \times 10^{-7}$ H $m^{-1}$ the magnetic permeability in vacuum, respectively. As a consequence of this pressure, the membrane is subjected to an elastic force [55]:

(5) $$F=\frac{\varepsilon \;E^{2}}{2}\;A\;2\pi \;r$$

where *A* is the surface of the membrane which the electromagnetic wave hits, while *r* represents the mean value of the internal cell radius. As a consequence of the membrane surface deformation caused by this force, the membrane’s electric potential is affected by local variation:

(6) $$\Delta \phi =E\;A=\sqrt{\frac{F_{el}\;A}{\pi \;\varepsilon \;r}}\Rightarrow d\phi =A\;dE$$

with the consequence to force the opening of ion channels for inflows and outflows. As such, since in our quest to reduce inflammation, we need to force ${\mathrm{Na}}^{+}$ outflow only, we must find the value of *E* and *B*. We remember that a pressure variation generates a change in the Gibbs free energy [52]:

(7) $$dG=V\;dp-S\;dT+\sum_{i}\mu_{i}\;dN_{i}=V\;\varepsilon \;E\;dE-S\;dT+\sum_{i}\mu_{i}\;dN_{i}$$

where *V* is the volume of the system considered, *S* is the entropy, $\mu_{i}$ represents the chemical potential of the i-th chemical species, and *N* stands for the number of particles which flow across the boundary of the system. Experimental results obtained in the last decade have always confirmed this approach [56].

Consequently, an incoming electromagnetic wave can alter the cell membrane potential ϕ, with a consequent change in the ion concentration on both sides of the membrane (Equation (1)).

## 3. Results

In this paper, we have established a connection between the energy characteristics of electromagnetic waves and the local pressure variation (radiation pressure) as well as the Gibbs free energy. Consequently, the biological effect of low-frequency electromagnetic waves can be explained by the membrane’s elastic response to radiation pressure, by changing the membrane electric potential, and consequently, inducing a change in ion concentration with related flows.

Indeed, considering that a human is an assortment of more than 200 different cell types for a total amount of $\left(3.7\pm 0.8\right)\times 10^{13}$ cells which perform a staggering variety of functions and have a diameter in the range of 10–100 µm, an applied 100 µT magnetic field component of an electromagnetic wave determines an effective value of an applied magnetic field of 70 µT, and a pressure in the order of 0.31–3.13 Pa, with a membrane electric potential variation of 0.04–4.17 mV.

This result must be compared with the normal membrane electric potential of −70 mV and that of cancer cells of around −10 mV. The results are in the order of 0.06–6.00% for normal cells and 0.04–7.00% for cancer cells.

Considering the definition of Gibbs free energy in Equation (3), it follows that

(8) $$\dot{Q}\;\tau =\Delta H-ZF\;\Delta \phi -2.3R\;T\;\Delta \mathrm{pH}$$

where *H* is the enthalpy, and $\dot{Q}=T\;\dot{S}$, where $\dot{S}$ is the entropy production rate in the environment and the heat results $Q=\dot{Q}\cdot \tau$, with τ characteristic time of the cell system in relation to heat transfer. Equation (8) highlights the mechanism of ELF-EMF by linking the quantity Δϕ+2.3RTΔpH/F to the characteristic time, τ, at a definite value of inflow energy, ΔH, and outflow heat power, $\dot{Q}$.

To evaluate the characteristic time, τ, the membrane heat transfer, can be considered by introducing the thermokinetic lumped model [57]. Indeed, the cell exchanges heat power with its environment, and this heat outflow is strictly related to the cell’s metabolism. The heat outflow can occur by convection with the fluids around the cell [58]:

(9) $$\dot{Q}=\rho_{cell}Vc_{cell}\frac{dT_{cell}}{dt}=\alpha A\left(T_{cell}-T\right)$$

where $\rho_{cell}$ and *V* are the cell’s mass density and volume, respectively, $c_{cell}$ is the cell’s specific heat, $T_{cell}$ is its temperature, α is the coefficient of convection, *A* is the cell’s surface area, which can change during the different cell development phases, and $T_{cell}-T$ is the temperature difference between the cell and its environment temperature. Consequently, the characteristic time τ results in [50]

(10) $$\tau =\frac{\rho_{cell}\;c_{cell}}{\alpha }\;\frac{V}{A}$$

resulting in a resonant process.

Consequently, at a resonant state, the heat outflow results in its maximum fluxes [59], as a consequence of the definition of resonance. Thus, the increase in heat outflow depolarises the cell membrane and restores normal conditions when the cell system is in an inflammation, cancer, or disease state.

The confirmation of the theoretical results of Equation (10) has been obtained by means of experimental evidence. This investigation involved the growth comparison of specific cancer cell lines when subjected to extremely low-frequency electromagnetic fields (ELF-EMFs) at their resonant frequencies, with the growth of untreated cells from the same lines. For each cell line, the resonant frequency was determined through the analysis of the average geometric parameters of the cells themselves, in accordance with theoretical findings. The ELF-EMF exposure system was constituted by two couples of coaxial coils, wound into a cylindrical frame (external radius: 0.08 m, distance among the couples of coaxial coils: 0.08 m). The external casing of the exposure system was constituted by a box-shaped magnetic metal shield to avoid any interference from the background magnetic field, in order to obtain the cells’ exposure just at their resonant frequencies, without external alterations of the signal. When treated, the exposed cells were inserted at the centre of the box-shaped structure within the incubator, while the control group of the same cell line (untreated cells) were inserted within the same incubator, without any shielding and exposure system. The results summarised in Table 1 show the calculated resonant frequency for each cancer cell line. The cells were exposed to their respective characteristic resonant frequencies, and the impact on their growth was compared to that of the untreated cells. It is worth noting the following:

- The experimental results obtained were repeatable, and the cell’s behaviour was consistent;
- The cancer growth rate is reduced by electromagnetic waves at the cell’s thermal resonant frequency;
- The phenomenon is selective with respect to the frequencies used, as it must be for a resonant process.

In Table 1, it is evident that the reduction in cancer growth depends on the frequency, which is influenced by the shapes of the cell lines. This suggests that the shape is related to the cell cycle phase. To explore potential applications in therapies, enhancing the ELF-EM effect could involve assessing the average volume/area ratio at different stages of the cell phase. These experimental results have been confirmed in other experiments, which were always conducted on cancer cells (spheroids included) [14,56,60,61,62].

**Table 1:** Experimental results obtained concerning the growth variation of some cancer cell lines after exposure to the calculated resonant frequencies under a sinusoidal magnetic field of 100 µT maximum amplitude [56,62].

Cell Line	Human Cancer	Frequency [Hz]	Growth Change [%]
A375P	melanoma cell line	31	−15
HT-29	colorectal adenocarcinoma	24	−19
GTL16	gastric cancer	14	−24
MCF7	breast cancer	5	−22
SKBR3	breast cancer	8	−18
MDA-MB-231	breast cancer	6	−18

## 4. Discussion

The interest in using electromagnetic fields for therapy dates back to the 19th century. Nikola Tesla was one of the first to suggest the potential therapeutic use of EMF in 1898 [63]. He observed that high-frequency currents seemed to pass through the body without causing pain or discomfort, indicating that tissues act as capacitors [63]. In 1932, Dr. Gustave Kolischer reported at a seminar by the American Congress of Physical Therapy in New York that Tesla’s high-frequency electrical currents were producing highly beneficial results in treating cancer, surpassing the outcomes achievable with regular surgery [64].

In 1922, the pioneering work of Russian histologist Alexander Gurvich and his wife revealed a groundbreaking discovery. They found that living cells, when separated by quartz glass, could communicate vital cell information. Through numerous experiments, they suggested that this information was transmitted by invisible light waves in a UV frequency spectrum that passed through quartz and was stopped by window glass [65]. This remarkable work provided the first documented evidence of biophotons, which are coherent light emitted by a biosystem. It laid the foundation for the development of later bioelectromagnetic therapy devices. Notably, it was not until the early 1960s that Leningrad State University successfully captured the mitogenic rays using sensitive photomultipliers [64,66].

In 1925, Georges Lakhovsky revolutionised the field by introducing the concept of resonance in ELF-EMF therapeutic effects [67]. He developed the Lakhovsky’s Radio-Cellulo-Oscillator, also known as the Multiple-Wave-Oscillator, which emitted low-frequency electromagnetic waves documented to deliver highly effective therapeutic results [64].

Abraham Liboff, a pioneering physicist, uncovered the electric field and geomagnetic ion cyclotron resonance, offering a more accurate explanation for the resonant interaction of static magnetic fields with endogenous AC electric fields in biological systems [8,68]. Recently, the crucial role of thermodynamics has been underscored in biomedicine by explaining how thermodynamic principles can shed light on the reasons for specific changes in cell and tissue structures that aid in the detection, identification, and staging of cancer [69]. It highlights the fundamental role of entropy in elucidating the different processes taking place in cancerous tissues at various scales and stages. Tissues can be likened to self-assembled and self-organised structures that arise under non-equilibrium conditions, resulting in entropy production and energy dissipation [50,70]. Moreover, their response to external stimuli is contingent on the intrinsic properties of these structures [12,69,71,72].

The approach developed here needs to be considered in this context. It is a thermodynamic approach that deals with open systems under non-equilibrium conditions, just like real cells. This approach is based on the fundamental conservation laws of thermodynamics: the conservation of mass, charge, and energy. Additionally, it takes into account the fundamental parameter of the cell’s membrane, which is the electric potential, as pointed out by Cone Jr. in 1969 [73,74,75]. Furthermore, this approach is built upon the thermal resonance effect and assesses irreversibility through the measurement of entropy.

Finally, this approach is easy to assess, based on fundamental laws of nature, and it has consistently yielded reproducible experimental results over the past decade.

## 5. Conclusions

ELF-EMFs are being successfully used in modern medicine to address certain health issues, especially those for which conventional medicine has not yet produced satisfactory results. This therapy offers a non-invasive future approach to treating the injury site, pain, and inflammation in different pathologies [76]. In this paper, we reported some of the possible therapeutic applications of ELF-EMFs in the treatment of various clinical conditions, focusing on biological endpoints such as pain relief, improved healing in skeletal trauma and cutaneous wounds, and containment of malignant tumour progression. The identification and exposition of tumour-specific ELF-EMFs have been suggested for containing cancer without any side effects or toxicity [77,78] even in patients with advanced cancer or inflammation. Existing data show that ELF-EMF exposure could extend patients’ survival time, limit cancer cell replication, stimulate apoptosis-like behaviour [79,80,81,82], and also impair angiogenesis in solid tumours [83,84,85].

The effects of ELF-EMF were analysed in association with standard therapies, assuming a possible synergistic effect [79,86,87]. Additionally, it was observed that they could revert the resistance to chemotherapy of cancer cells *in vitro* [88,89]. Other EMF treatments were aimed to improve chemotherapy side effects [90], as well as enhance the well-being of terminally ill patients [91].

There is evidence that EMFs effectively reduce cellular inflammation and pain; indeed, ELF-EMFs have been shown to impact pain and inflammation by modulating G-protein coupling receptors (GPCRs), lowering cyclooxygenase-2 (Cox-2) and nuclear factor kappa B (NF-κB) necessary to induce inflammatory mediators [92]. ELF-EMFs have the ability to shift the transition from a pro-inflammatory to an anti-inflammatory state, upregulate nitric oxide synthase (NOS) activity, and downregulate Cox-2 expression and Prostaglandin E2 (PGE-2) production, which are involved in the modulation of inflammatory reactions [9,93].

Bone repair stimulation is one of the stronger and better-documented therapeutic effects of EMFs. It is widely used to aid in the healing of fractures by enhancing the proliferation and differentiation of osteoblasts [94,95]; indeed magneto-therapy devices have been successfully used as adjunctive therapy for the treatment of delayed and non-union fractures, fresh fractures, and chronic wounds [96].

In cases of traumatic spinal cord injury where the disruption of axonal transmission of signals leads to neuro-inflammation, neurodegeneration, and cytotoxicity, an alternative method to electrically activate spinal circuits is the application of an external ELF-EMF [97]. In skin and soft tissue healing, ELF-EMF exposure is effective in vascularisation, reduction of wound depth by granulation tissue, and reduced inflammatory cell migration and infiltration. In a cutaneous wound model *in vitro*, ELF-EMFs were able to enhance migration and proliferation of keratinocytes from the periphery of the wound, switching from the inflammatory phase to the final repair phase and inducing more rapid healing of the wound [98].

In conclusion, although different types of ELF-EMFs are now effectively used in medicine (some examples of possible uses of ELF-EMFs in medicine are summarised from the literature in Table 2), there is still a lack of understanding regarding the basic interaction mechanism between ELF-EMFs and cells. Here, we provide a thermodynamic approach to explain the effects of ELF-EMFs on energy, mass, and ionic flows through the cell membranes and the resultant changes in cell behaviour.

The lipid bilayer membrane of a living cell has different specific ion concentrations on each side, regulated by the electro-diffusion caused by the electrochemical gradient of the negative membrane potential (around −70 mV). The cell’s functions are controlled by membrane proteins which undergo conformational modifications due to voltage-responsive transduction mechanisms. So, ELF-EMFs’ interactions with the membrane are interesting for possible future therapeutic support to some diseases, e.g., cancer and inflammation. Furthermore, electromagnetic waves’ interactions with cells are shown to be frequency-dependent due to their resonant nature.

## Figures and Tables

**Table 2:** Some examples of evidence of possible uses of ELF-EMFs in medicine from the literature [4].

Disease	Frequency [Hz]	Key Finding	Ref.
Arthritis	60	Reduction in pain and inflammation	[99]
Back pain	64	Statistically significant for reducing pain	[100]
Cancer ${*}^{1}$	0.1–($114\times 10^{3}$)	Significant decrease in size of tumour ${*}^{2}$	[81]
Carpal tunnel	20	Statistically significant pain reduction ${*}^{3}$	[101]

${*}^{1}$ breast, colon, and prostate tumours; ${*}^{2}$ with tumour-specific frequencies; ${*}^{3}$ short- and long-term.

## Data Availability

All the references for the data are quoted in the paper.
